# Supplementary material for: Carbon Dioxide Tornado-Type Atmospheric-Pressure-Plasma-Jet-Processed rGO-SnO2 Nanocomposites for Symmetric Supercapacitors
Source: Materials (Basel). 2021 May 24;14(11):2777. doi: 10.3390/ma14112777 (PMC8197281; doi:10.3390/ma14112777)
Supplement: Supplementary file 1 [file materials-14-02777-s001.zip › materials-1234335-SUP-conversion.pdf]

materials

Supplementary Materials

Carbon Dioxide Tornado-Type Atmospheric-Pressure-Plasma-Jet-Processed rGO-SnO_2 Nanocomposites for Symmetric Supercapacitors

Jung-Hsien Chang [1,2,†], Song-Yu Chen [3,†], Yu-Lin Kuo [3,*], Chii-Rong Yang [4] and Jian-Zhang Chen [1,2,5,*]

1 Graduate Institute of Applied Mechanics, National Taiwan University, Taipei City 10617, Taiwan; r08543006@ntu.edu.tw
2 Advanced Research Center for Green Materials Science and Technology, National Taiwan University, Taipei City 10617, Taiwan
3 Department of Mechanical Engineering, National Taiwan University of Science and Technology, Taipei City 10607, Taiwan; D10803004@gapps.ntust.edu.tw
4 Department of Mechatronic Engineering, National Taiwan Normal University, Taipei City 10610, Taiwan; ycr@ntnu.edu.tw
5 Innovative Photonics Advanced Research Center (i-PARC), National Taiwan University, Taipei City 10617, Taiwan
* Correspondence: ylkuo@mail.ntust.edu.tw (Y.-L.K.); jchen@ntu.edu.tw (J.-Z.C.)
† J.-H.C. and S.-Y.C. contributed equally to this work.

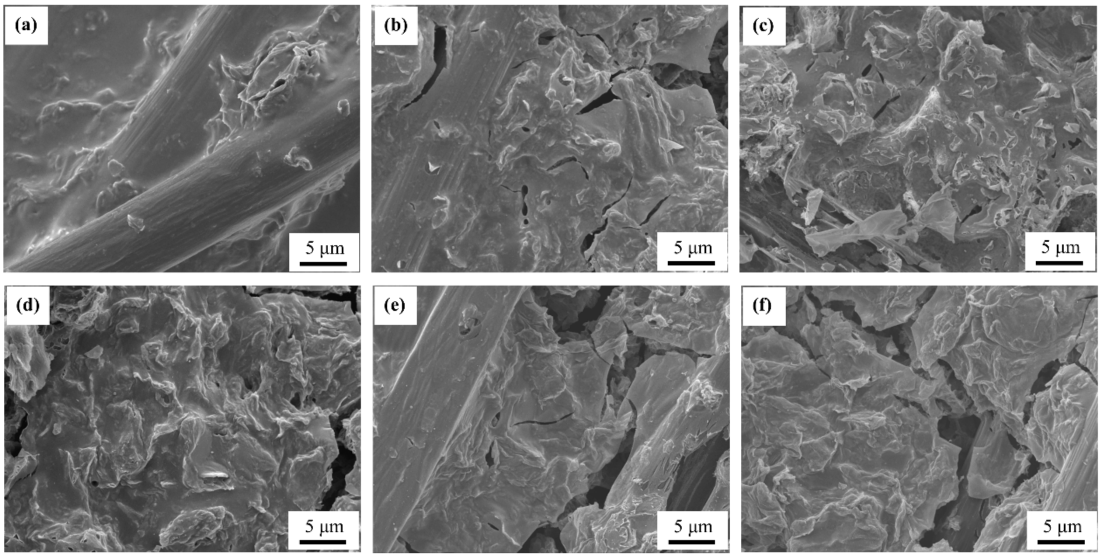

Figure S1. SEM images (3,000×) of rGO–SnO_2 electrodes scanned (**a**) zero, (**b**) one, (**c**) three, (**d**) five, (**e**) seven, and (**f**) nine times using CO_2 APPJ.

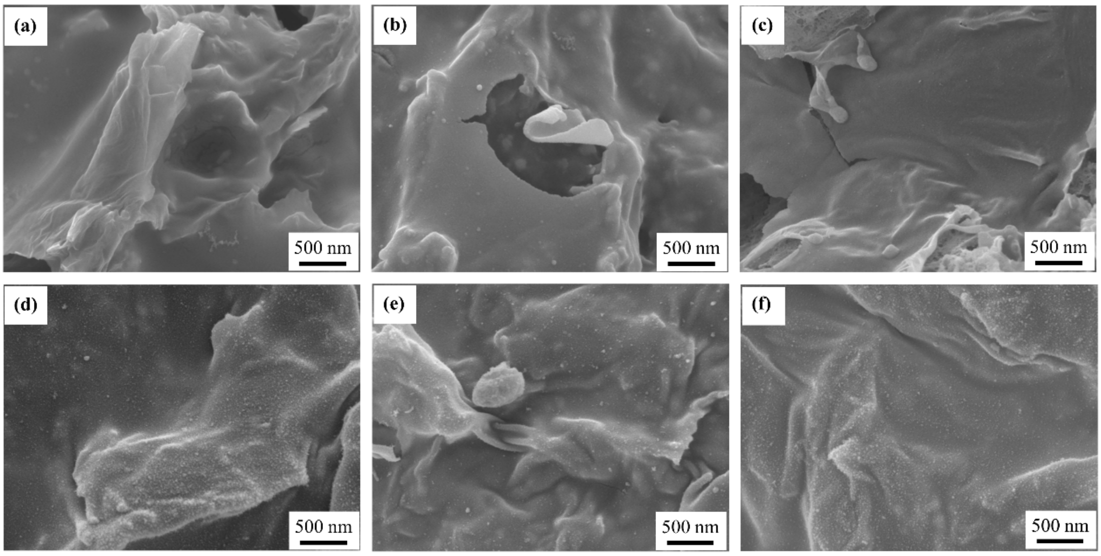

Figure S2. SEM images (30,000×) of rGO–SnO_2 electrodes scanned (**a**) zero, (**b**) one, (**c**) three, (**d**) five, (**e**) seven, and (**f**) nine times using CO_2 APPJ.

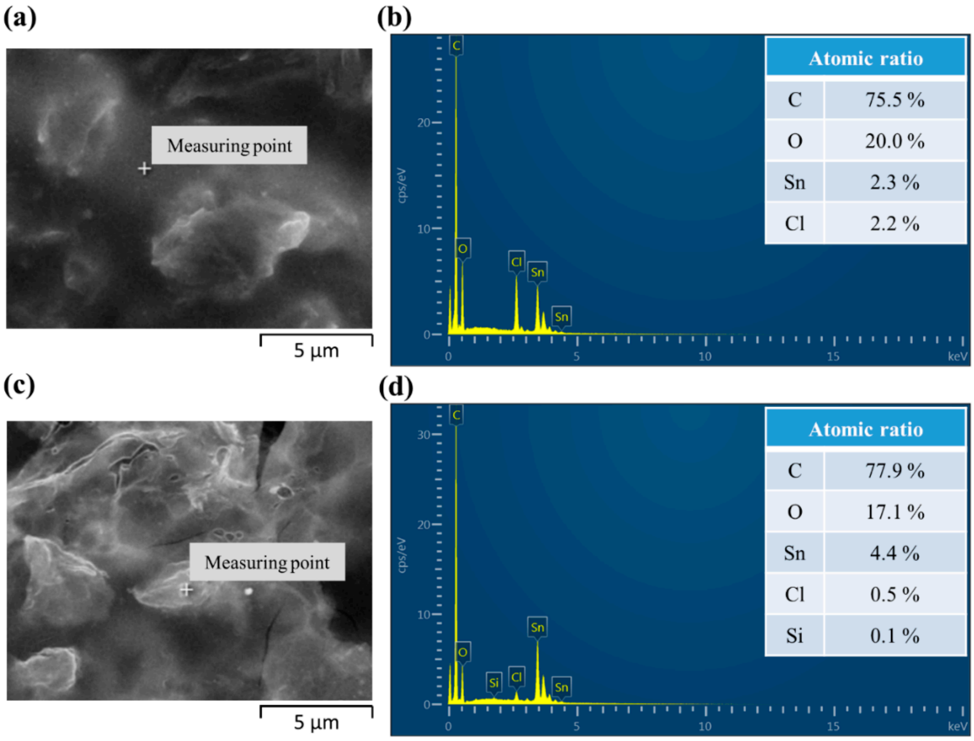

Figure S3. Energy-dispersive spectroscopy (EDS) analysis of the electrodes: (**a**) The measuring points of as-deposited electrode. (**b**) EDS analysis of as-deposited electrode. (**c**) The measuring points of seven-times APPJ-scanned electrode. (**d**) EDS analysis of seven-times APPJ scanned electrode.

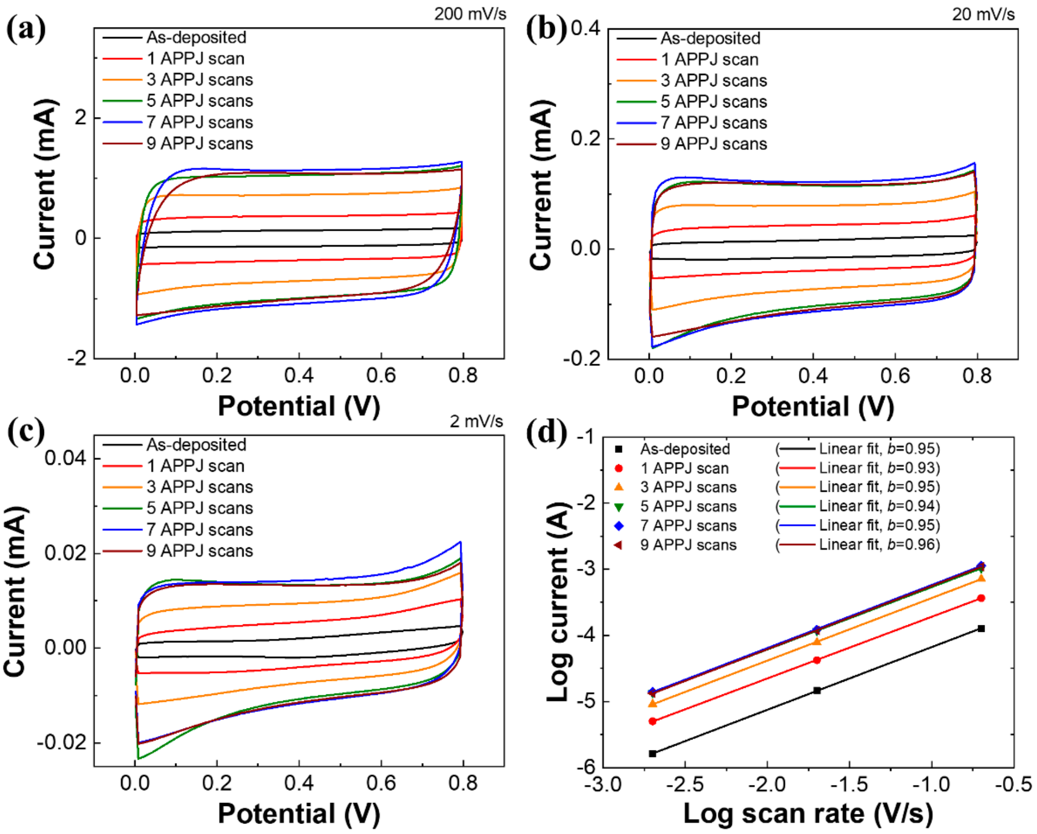

Figure S4. CV of bare carbon cloth SCs (without rGO–SnO_2) under potential scan rates of (**a**) 200 mV, (**b**) 20 mV, and (**c**) 2 mV. (**d**) Logarithm of currents and scan rates.

Table S1. Areal capacitance of bare carbon cloth SCs (without rGO–SnO_2).

**Areal capacitance (mF/cm²)**			
**Number of APPJ scans**	**Potential scan rate (mV/s)**		
	**2**	**20**	**200**
**0**	0.61	0.52	0.43
**1**	1.57	1.37	1.20
**3**	2.87	2.57	2.35
**5**	4.28	3.76	3.31
**7**	4.48	4.02	3.48
**9**	4.24	3.80	3.18

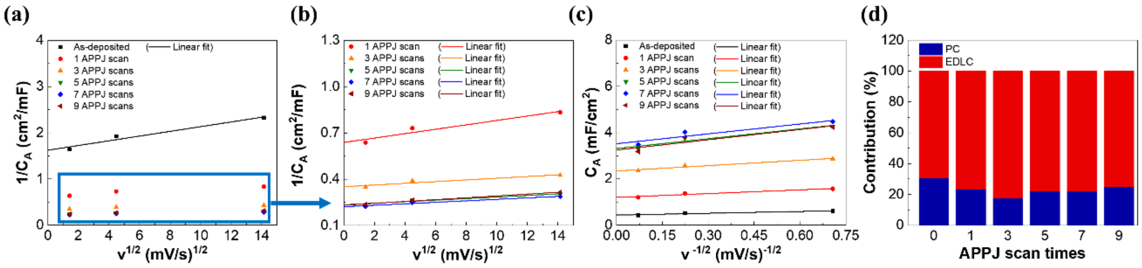

Figure S5. Trasatti plots of bare carbon cloth electrode SCs: (**a**,**b**) $1/C_A$ vs. $v^{1/2}$, (**c**) C_A vs. $v^{-1/2}$, and (**d**) capacitive contribution.

Table S2. Capacitive contribution of carbon cloth SCs.

Number of APPJ scans	C_{total} (mF/cm^2)	C_{in} (mF/cm^2)	C_{out} (mF/cm^2)	Capacitive contribution (PC:EDLC) (%)
**0**	0.62	0.19	0.43	30.6:69.4
**1**	1.57	0.37	1.20	23.6:76.4
**3**	2.85	0.51	2.34	17.9:82.1
**5**	4.27	0.96	3.31	22.5:77.5
**7**	4.52	1.00	3.52	22.1:77.9
**9**	4.32	1.08	3.24	25.0:75.0

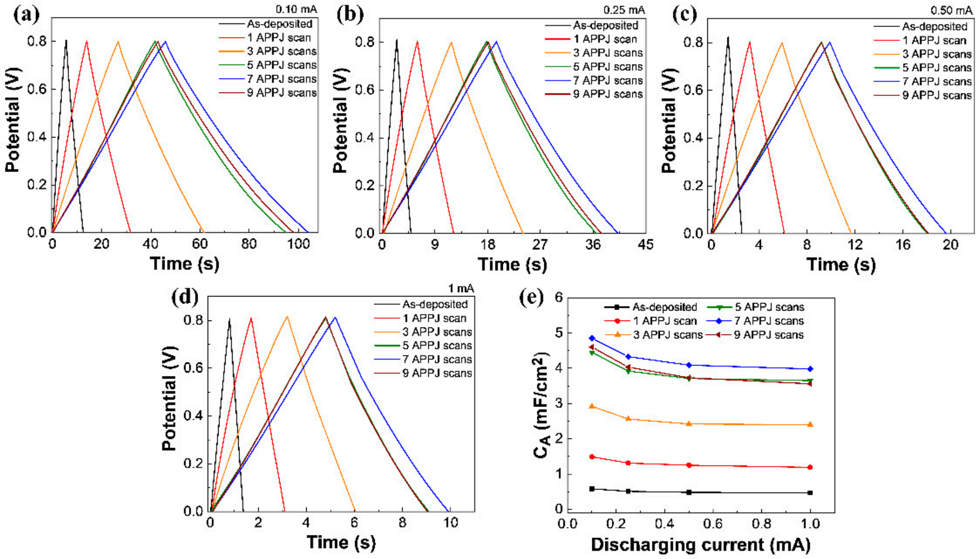

Figure S6. GCD of carbon cloth SC under constant current of (**a**) 0.10 mA, (**b**) 0.25 mA, (**c**) 0.50 mA, and (**d**) 1 mA. (**e**) Areal capacitances calculated based on GCD results.

Table S3. Areal capacitance of carbon cloth SCs calculated by GCD.

Areal capacitance (mF/cm^2)				
**Number of APPJ scans**	**Discharging current (mA)**			
	**0.10**	**0.25**	**0.50**	**1**
**0**	0.58	0.51	0.48	0.47
**1**	1.49	1.31	1.25	1.19
**3**	2.92	2.56	2.42	2.40
**5**	4.45	3.92	3.71	3.65
**7**	4.85	4.33	4.09	3.98
**9**	4.60	4.03	3.73	3.56
